# Vasopressor use following traumatic injury – A single center retrospective study

**DOI:** 10.1371/journal.pone.0176587

**Published:** 2017-04-27

**Authors:** Mathieu Hylands, Marie-Pier Godbout, Sandeep K. Mayer, William D. Fraser, Alain Vanasse, Marc-André Leclair, Alexis F. Turgeon, François Lauzier, Emmanuel Charbonney, Vincent Trottier, Tarek S. Razek, André Roy, Frédérick D’Aragon, Emilie Belley-Côté, Andrew G. Day, Soazig Le Guillan, Robert Sabbagh, François Lamontagne

**Affiliations:** 1 Division of General Surgery, Université de Sherbrooke, Sherbrooke, Québec, Canada; 2 Centre de recherche du Centre hospitalier universitaire de Sherbrooke, Sherbrooke, Québec, Canada; 3 Department of Obstetrics and Gynecology, Université de Sherbrooke, Sherbrooke, Québec, Canada; 4 Department of Family Medicine and Emergency Medicine, Université de Sherbrooke, Sherbrooke, Québec, Canada; 5 Department of Medicine, Université de Sherbrooke, Sherbrooke, Québec, Canada; 6 Department of Anesthesiology and Critical Care, Université Laval, Québec, Québec, Canada; 7 Centre de recherche du Centre hospitalier universitaire de Québec, Québec, Québec, Canada; 8 Department of Medicine, Université Laval, Québec, Québec, Canada; 9 Department of Critical Care, Université de Montréal, Montréal, Québec, Canada; 10 Centre de recherche de l’hôpital du Sacré-Cœur de Montréal, Montréal, Québec, Canada; 11 Department of General Surgery, Université Laval, Québec, Québec, Canada; 12 Department of General Surgery/Trauma Surgery, MUHC Montreal General Hospital, Montreal, Quebec, Canada; 13 Department of Physiatry, Université de Montréal, Montréal, Québec, Canada; 14 Centre de recherche du Centre Hospitalier Universitaire de Montréal, Montréal, Québec, Canada; 15 Department of Anesthesiology, Université de Sherbrooke, Sherbrooke, Québec, Canada; 16 Department of Community Health and Epidemiology, Queen’s University, Kingston, Ontario, Canada; 17 Division of Traumatology/General Surgery, Sacré-Coeur Hospital of Montreal, Montreal, Canada; 18 Department of Urology, Université de Sherbrooke, Sherbrooke, Québec, Canada; Cedars-Sinai Medical Center, UNITED STATES

## Abstract

**Objectives:**

Vasopressors are not recommended by current trauma guidelines, but recent reports indicate that they are commonly used. We aimed to describe the early hemodynamic management of trauma patients outside densely populated urban centers.

**Methods:**

We conducted a single-center retrospective cohort study in a Canadian regional trauma center. All adult patients treated for traumatic injury in 2013 who died within 24 hours of admission or were transferred to the intensive care unit were included. A systolic blood pressure <90 mmHg, a mean arterial pressure <60 mmHg, the use of vasopressors or ≥2 L of intravenous fluids defined hemodynamic instability. Main outcome measures were use of intravenous fluids and vasopressors prior to surgical or endovascular management.

**Results:**

Of 111 eligible patients, 63 met our criteria for hemodynamic instability. Of these, 60 (95%) had sustained blunt injury and 22 (35%) had concomitant severe traumatic brain injury. The subgroup of patients referred from a primary or secondary hospital (20 of 63, 32%) had significantly longer transport times (243 vs. 61 min, p<0.01). Vasopressors, used in 26 patients (41%), were independently associated with severe traumatic brain injury (odds ratio 10.2, 95% CI 2.7–38.5).

**Conclusions:**

In this cohort, most trauma patients had suffered multiple blunt injuries. Patients were likely to receive vasopressors during the early phase of trauma care, particularly if they exhibited signs of neurologic injury. While these results may be context-specific, determining the risk-benefit trade-offs of fluid resuscitation, vasopressors and permissive hypotension in specific patients subgroups constitutes a priority for trauma research going forwards.

## Background

Worldwide, traumatic injuries caused 5.1 million deaths in 2010, [1] a figure which exceeds mortality from malaria, HIV-AIDS and tuberculosis combined. [2] This number is expected to grow to 8 million by 2020. [3] Efforts aimed at reducing trauma morbidity and mortality are both timely and warranted.

The early management of trauma patients is critical in influencing outcome. [4, 5] The main causes of death following trauma are central nervous system injuries and hemorrhage. [6, 7] Accordingly, the goals of early resuscitation are to preserve cerebral perfusion and control bleeding. Reconciling these two objectives constitutes a challenge, since efforts to restore tissue perfusion may exacerbate bleeding. [8] In a landmark clinical trial, hypotensive patients with penetrating torso trauma were more likely to be discharged alive from hospital when fluid resuscitation was withheld until arrival to the operating room. [8] These results are concordant with data from a subsequent 90-patient trial of fluid use during emergent trauma surgery, where a mean arterial pressure (MAP) target of 50 mmHg vs. 65 mmHg significantly decreased blood product use without increasing 30-day mortality. [9] Accordingly, recent trauma guidelines have incorporated restrictive fluid strategies, referred to as permissive hypotension, into their recommendations. [10, 11] However, as pointed out in two systematic reviews, [12, 13] the safety of permissive hypotension remains uncertain. This may be particularly true among patients who have suffered a traumatic brain injury (TBI), in whom hypotension is associated with increased mortality. [8, 9, 14–16]

Used as fluid-sparing adjuncts to resuscitation, vasopressors can complement resuscitative measures by correcting hypotension without diluting clotting factors or increasing the risk for tissue edema. [17] While vasopressors have been associated with increased mortality in observational studies, [18, 19] residual bias likely confounds these associations. In a recent 78-patient clinical trial, vasopressor use was associated with reduced fluid administration. This trial, which was stopped early due to low recruitment, was not powered to capture an effect on mortality. [20] In the absence of solid evidence, experts recommend limiting vasopressor therapy to situations where patients are unresponsive to fluid therapy. [10, 11]

The objective of this study was to describe the early hemodynamic management of trauma patients outside densely populated urban centers, particularly with regards to fluid administration and vasopressor use. We also aimed to describe trajectories of care to quantify the potential duration of resuscitative measures that occur before surgical or endovascular management.

## Methods

We conducted a retrospective study at the *Centre Intégré Universitaire de Santé et de Services Sociaux (CIUSSS) de l'Estrie—Centre Hospitalier Universitaire de Sherbrooke (CHUS)*, a university hospital and regional (level II) trauma center in Québec. This institution delivers definitive trauma care in a catchment area covering a population of 500,000.

### Eligibility

We reviewed the medical records of consecutive patients who presented with an admission diagnosis of traumatic injury from January 1^st^ to December 31^st^, 2013. This information is classified in hospital discharge summaries and follows the International Statistical Classification of Diseases-10 index. We included patients who were admitted to the intensive care unit (ICU) or who died within 24 hours of admission to the *CIUSSS de l’Estrie—CHUS*. In this center, any patient at risk of hemodynamic instability is admitted to the ICU. We excluded patients with "do not resuscitate" orders documented within the first six hours of care and those who had sustained a traumatic injury more than 24 hours prior to admission.

Eligible patients were divided in two categories on the basis of hemodynamic status. Patients were considered hemodynamically unstable if they exhibited at least one of the following criteria: MAP below 60 mmHg, systolic blood pressure (SBP) below 90 mmHg, received vasopressors or received ≥2 L of intravenous fluids (crystalloid, colloid or blood products) during the early resuscitation period.

### Data collection

We defined the early resuscitation period as the period from the arrival at a hospital (the *CIUSSS de l'Estrie—CHUS* or one of five primary or secondary referring hospitals) to the time of initial surgical or endovascular management. For patients who did not require surgical or endovascular procedures, we arbitrarily determined that the early resuscitation period ended six hours after arrival at the *CIUSSS de l'Estrie—CHUS*. We did not collect data on prehospital management since hemodynamic resuscitation interventions (i.e., vasopressors or intravenous fluids) are not administered by emergency medical technicians in Québec and therefore could not have been administered outside a hospital setting. During this early resuscitation period, invasive monitoring devices such as intracranial pressure monitors are not used. Clinical decision-making is therefore guided by clinical assessment.

Before initiating the study, we defined three resuscitation strategies: liberal intravenous fluid use, vasopressor use, and neither liberal fluid nor vasopressor use. Because Advanced Trauma Life Support guidelines recommend a trial of 1–2 L of crystalloid for hemodynamically unstable patients, we defined liberal intravenous fluid use as the administration of more than 2 L of crystalloid, colloid and/or blood products. [11] We defined vasopressor use as the administration of any vasoconstrictive agent (i.e., ephedrine, phenylephrine, norepinephrine, epinephrine, vasopressin, and dopamine). According to these definitions, liberal intravenous fluid therapy and vasopressor use are not mutually exclusive strategies. Patients who were neither exposed to more than 2 L of intravenous fluids nor to vasopressors and had a documented SBP under 90 mmHg or MAP under 60 mmHg constitute a distinct subgroup of patients.

Other variables of interest were documented blood pressure measurements during the early resuscitation period, baseline patient characteristics, Injury Severity Score (ISS), Prehospital Trauma Index (PTI), [21] GCS, trajectories of care (time between injury and arrival at the *CIUSSS de l'Estrie—CHUS*, time between injury and surgery or angiography), documented prescriptions for blood pressure targets, and clinical outcomes (ICU and hospital length of stay, discharge disposition, and in-hospital mortality). Severe TBI was defined as a presenting GCS ≤8. We performed a *post-hoc* review of computed tomography (CT) scan results for patients presenting with a severe TBI, in order to identify the proportion of these patients that had a confirmed intracranial lesion.

Twenty percent of eligible medical records were reviewed in duplicate by independent reviewers. We measured the chance-corrected inter-rater agreement (i.e., kappa) for the categorization of the resuscitation strategy. We predetermined that if kappa was greater than 0.6 (consensus definition for "substantial agreement"), [22] a single investigator would complete the remainder of the data collection. Chance corrected inter-rater agreement was 0.83 for liberal intravenous fluid use and 1.00 for both vasopressor use and use of neither liberal intravenous fluids nor vasopressors.

The Research Ethics Board of the *CIUSSS de l'Estrie—CHUS* approved this study.

### Statistical analyses

We present continuous variables as means (standard deviations [SD]) or medians (first, third quartiles) as appropriate and categorical variables as counts and proportions.

We conducted between-group comparisons using Fisher’s exact test for proportions and Student’s independent T-test or Wilcoxon Rank-Sum test, as appropriate, for continuous variables.

We used a multivariable logistic regression model to explore associations between age, ISS, severe TBI (i.e., GCS of 8 or less), and pre-existing chronic hypertension (i.e., independent variables) and vasopressor use (dependent variable). Independent variables were selected *a priori*, based on clinical significance and data availability, and were introduced into the model simultaneously. In a sensitivity analysis, we repeated the analysis using backward stepwise selection.

Two-sided p-values less than 5% were considered significant without correction for multiple testing. Analyses were conducted using SAS version 9.4 (SAS Institute Inc., Cary, NC, USA).

## Results

**Fig 1:**
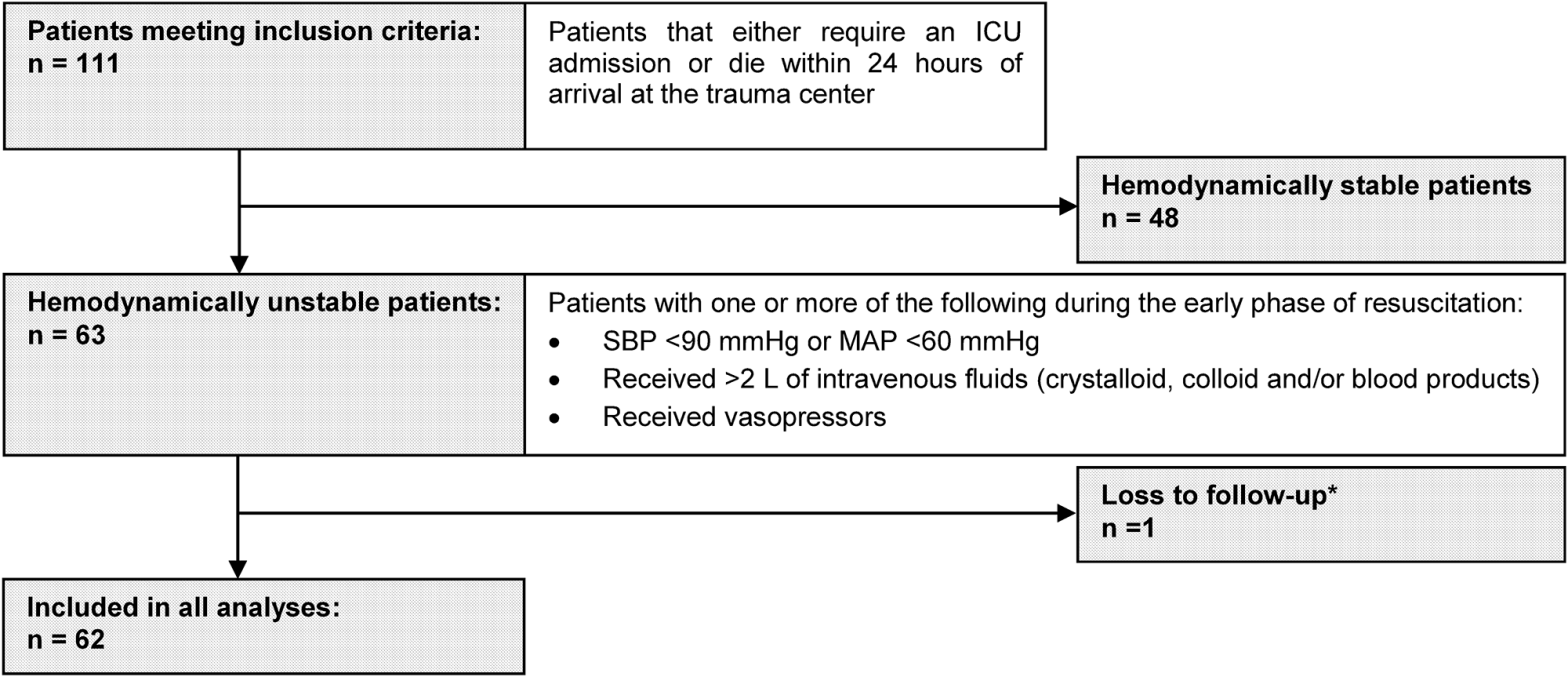
Patient flow diagram. *One patient was transferred to another hospital after the initial phase of resuscitation and could not provide outcome data. ICU—Intensive care unit, SBP—Systolic blood pressure, MAP—Mean arterial pressure.

Overall, 111 patients fulfilled the eligibility criteria, of whom 63/111 (57%) were hemodynamically unstable during the early resuscitation period. Twenty-nine patients (29/111, 26%) were transferred from a primary or secondary referring hospital and 107/111 (96%) had sustained a blunt injury. [Table 1] [Fig 1]

**Table 1:** Baseline characteristics by hemodynamic status.

		Hemodynamic status				
		Unstable		Stable		p-value
		n	value	n	value	
**Age**		63	49.4±21.4	48	53.2±22.0	0.37
**Age <45**			33 (52.4%)		29 (60.4%)	0.44
**Sex**	**Male**		48 (76.2%)		30 (62.5%)	0.14
**Type of trauma**	**Blunt**		60 (95.2%)		47 (97.9%)	0.63
**Prehospital Trauma Index**		46	4.9±3.8	37	2.5±3.2	<0.01
**Injury Severity Score**		63	21.7±12.7	47	17.8±10.4	0.08
**Glasgow Coma Scale**		63	10.7±4.5	48	12.5±3.8	0.02
Severe TBI*			22 (34.9%)		11 (22.9%)	0.21
Intoxication**			18 (28.6%)		12 (25.0%)	0.83
**Past medical history**	**Hypertension**		18 (28.6%)		14 (29.2%)	
	**Chronic renal failure**		4 (6.3%)		2 (4.2%)	
	**Coronary artery disease**		9 (14.3%)		6 (12.5%)	
	**Peripheral vascular disease**		3 (4.8%)		3 (6.3%)	
	**Congestive heart failure**		0 (0.0%)		1 (2.1%)	
	**Diabetes**		10 (15.9%)		6 (12.5%)	
**Transfer from primary or secondary referring hospital**			20 (31.8%)		9 (18.8%)	0.13

Categorical variables are presented as count (%), continuous variables as mean±SD. p-values comparing the eligible to non-eligible patients are by Fisher's exact test and by Student's t-test for categorical and continuous variables respectively.

*TBI: traumatic brain injury; severe TBI is defined as Glasgow Coma Scale ≤8

**illicit drugs or alcohol

### Hemodynamically unstable patients

Hemodynamically unstable patients were, on average, 49.4 (21.4) years old and 48/63 (76%) were male. Blunt trauma was the mechanism of injury for 60/63 (95%) patients and the mean ISS for hemodynamically unstable patients was 21.7 (12.7), consistent with severe injury. [23] Severe TBI was present in 22/63 (35%) cases. Patients meeting criteria for hemodynamic instability had a higher average PTI (4.9 vs. 2.5, p<0.01) and lower GCS (10.7 vs. 12.5, p = 0.02) than hemodynamically stable patients. [Table 1] Eighteen (18/63, 29%) hemodynamically unstable patients underwent a surgical intervention and five (5/63, 8%), an endovascular procedure while one patient underwent both. The other hemodynamically unstable patients (39/63, 62%) were stabilized using medical interventions only.

The 22 unstable patients with a GCS ≤8 had higher injury scores than patients with a GCS >8. [Table 2] The median [Q1, Q3] duration of mechanical ventilation for patients with an initial CGS ≤8 was 5 [2, 9] days. Only 2/22 patients (9%) were extubated on the day of presentation and 4/22 (18%) on the following day. Median neuro SOFA scores [24] for survivors that remained in the ICU were 3 [2, 4] on day 2 (n = 21) and 3 [2, 3] on day 3 (n = 17). In 18/22 patients, final CT imaging reports confirmed the presence of an intracranial traumatic injury (i.e. subdural hematoma, epidural hematoma, subarachnoid hemorrhage, parenchymal contusions and/or parenchymal hemorrhage).

**Table 2:** Baseline characteristics of hemodynamically unstable patients with and without TBI*.

		Severe traumatic brain injury				
		Present (n = 22)		Absent (n = 41)		p-value
		n	value	n	value	
**Age**		22	49.4±20.2	41	49.5±22.2	0.99
**Age <45**			11 (50.0%)		19 (46.3%)	0.80
**Sex**	**Male**		16 (72.7%)		32 (78.0%)	0.76
**Type of trauma**	**Blunt**		22 (100.0%)		38 (92.7%)	0.55
**Prehospital Trauma Index**		18	7.6±3.3	28	3.1±3.0	<0.01
**Injury Severity Score**		22	26.9±14.2	41	18.9±10.1	0.01
**Glasgow Coma Scale**		22	5.1±1.8	41	13.6±1.9	<0.01
**Lowest MAP**		22	56.7±11.4	41	59.0±15.4	0.54
**TBI confirmed on CT**			18 (81.8%)		-	-
Intoxication**			9 (40.9%)		9 (22.0%)	0.15
**Past medical history**	**Hypertension**		6 (27.3%)		12 (29.3%)	
	**Chronic renal failure**		1 (4.5%)		3 (7.3%)	
	**Coronary artery disease**		4 (18.2%)		5 (12.2%)	
	**Peripheral vascular disease**		1 (4.5%)		2 (4.9%)	
	**Congestive heart failure**		0 (0.0%)		0 (0.0%)	
	**Diabetes**		4 (18.2%)		6 (14.6%)	
**Transfer from primary or secondary referring hospital**			6 (27.3%)		14 (34.1%)	0.77

Categorical variables are presented as count (%), continuous variables as mean±SD. p-values comparing the hemodynamically unstable patients with and without severe TBI are by Fisher's exact test and by Student's t-test for categorical and continuous variables respectively

*TBI: traumatic brain injury; severe TBI is defined as Glasgow Coma Scale ≤8

**illicit drugs or alcohol

### Resuscitation strategies

Among hemodynamically unstable patients, 46/63 (73%) received >2 L of intravenous fluids. Twenty-six patients (26/63, 41%) received vasopressors and 16/63 (25%) received both >2 L of intravenous fluids and vasopressors. Seven (7/63, 11%) received ≤2 L of intravenous fluids and no vasopressors. Amongst the subgroup of patients with a GCS ≤8, 13/22 (59%) patients received >2 L of intravenous fluids, 16/22 (73%) received vasopressors and 9/22 (41%) received both >2 L of fluids and vasopressors. Two patients (2/22, 9%) with GCS ≤8 received <2 L of fluids and no vasopressors.

Combining all fluids administered during the early resuscitation period, hemodynamically unstable patients received a median of 2.5 (1.3, 3.6) L and there were no significant differences in total fluid volume between patients who did and did not receive vasopressors. When vasopressors were initiated, patients had received a median of 1.0 (0.1, 1.9) L of intravenous fluids. There was no difference in the volume of blood products administered to patients that did or did not receive vasopressors (median: 0 [0, 323] mL for the vasopressor group vs. 0 [0, 568] mL for the no vasopressor group; p = 0.85). [Table 3] Of patients that received vasopressors, 12/26 (46%) received blood products compared to 16/37 (43%) patients that did not receive vasopressors.

**Table 3:** Fluid volumes (mL) administered during the initial phase of resuscitation*.

	Crystalloid/Colloid**	p	Blood Products	p	All Fluids	p
**VP + (n = 26)**	2010 (1101, 3399)	0.68	0 (0, 323)	0.85	2200 (1101, 4195)	0.69
**VP—(n = 37)**	2305 (1510, 3255)		0 (0, 568)		2588 (1512, 3580)	
**Overall (n = 63)**	2239 (1275, 3393)		0 (0, 568)		2515 (1275, 3581)	

Reported as median (Q1, Q3) with p-values from Wilcoxon Rank-Sum test. VP +: patients treated with vasopressors; VP—: patients treated without vasopressors

*Time period between injury and arrival to OR or angiography, or 6 hours (for patients that underwent neither)

**Three patients received colloids. Total volume of colloid per patient was ≤500 mL.

Patients treated with vasopressors had higher PTI, were more likely to have signs of severe TBI and had a trend towards shorter time to surgery than patients treated without vasopressors. Age, sex, ISS and the likelihood of being transferred from a referring hospital were similar between patients treated with and without vasopressors. [Table 4]

**Table 4:** Baseline characteristics of unstable patients treated with and without vasopressors.

		Vasopressor use				
		Yes (n = 26)		No (n = 37)		p-value
		n	value	n	value	
**Age**		26	54.4±21.6	37	45.9±20.8	0.12
**Age <45**			11 (42.3%)		19 (51.4%)	0.61
**Sex**	**Male**		20 (76.9%)		28 (75.7%)	1.00
**Type of trauma**	**Blunt**		23 (88.5%)		37 (100.0%)	0.07
**Prehospital Trauma Index**		18	6.7±4.0	28	3.7±3.3	0.01
**Injury Severity Score**		26	23.0±15.5	37	20.7±9.3	0.50
**Glasgow Coma Scale**		26	7.8±4.3	37	12.7±3.4	<0.01
Severe TBI*			16 (61.5%)		6 (16.2%)	<0.01
**Lowest MAP**		26	55.5±17.0	37	60.2±11.5	0.20
Intoxication**			10 (38.5%)		8 (21.6%)	0.17
**Past medical history**	**Hypertension**		9 (34.6%)		9 (24.3%)	
	**Chronic renal failure**		2 (7.7%)		2 (5.4%)	
	**Coronary artery disease**		5 (19.2%)		4 (10.8%)	
	**Peripheral vascular disease**		1 (3.8%)		2 (5.4%)	
	**Congestive heart failure**		0 (0.0%)		0 (0.0%)	
	**Diabetes**		4 (15.4%)		6 (16.2%)	
**Transfer from primary or secondary referring hospital**			8 (30.8%)		12 (32.4%)	1.00
**Time to surgical or angiographic intervention (hours)**		12	1.7 [0.8, 3.3]	12	2.9 [2.0, 5.0]	0.05

Categorical variables are presented as count (%), continuous variables as mean±SD or median [Q1, Q3]. p-values comparing the hemodynamically unstable patients with and without vasopressor use are by Fisher's exact test and by Student's t-test for categorical and continuous variables respectively. Time to surgical or angiographic intervention compared using Wilcoxon Rank-Sum test.

*TBI: traumatic brain injury; severe TBI is defined as Glasgow Coma Scale ≤8

**illicit drugs or alcohol

On regression analysis, severe brain injury was the only independent variable associated with vasopressor use during the early resuscitation period (OR 10.2, CI 2.7–38.5). [Table 5] Patients receiving both liberal fluids and vasopressors were more likely to have a severe TBI than those receiving only liberal fluids (56.3% vs. 13.3%, p<0.01). Blood pressure targets were explicitly documented for 24/63 (38%) hemodynamically unstable patients, more frequently among patients who received vasopressors (65% vs. 19%, p<0.01).

**Table 5:** Predictors of vasopressor use in hemodynamically unstable patients.

Predictors	Univariate model		Multivariable model		Selected model*	
	OR (95% CI)	p-value	OR (95% CI)	p-value	Odds Ratio	p-value
Age (per decade)	1.21 (0.95–1.55)	0.12	1.26 (0.90–1.77)	0.18	1.29 (0.97–1.71)	0.07
Injury Severity Score	1.02 (0.98–1.06)	0.45	0.99 (0.94–1.04)	0.75		
Chronic hypertension	1.65 (0.55–4.96)	0.38	1.13 (0.25–5.15)	0.87		
Severe TBI**	8.27 (2.54–26.86)	<0.01	10.16 (2.68–38.53)	<0.01	9.44 (2.72–32.71)	<0.01

An odds ratio >1 indicates the predictor is associated with a higher rate of vasopressor use during the initial phase of resuscitation. OR: odds ratio; CI: confidence interval; TBI: Traumatic brain injury

* Model selected using backward stepwise selection with p<0.15

** Severe TBI defined as Glasgow Coma Scale ≤8

### Trajectories of care

Among hemodynamically unstable patients, 20/63 (32%) initially received care at a primary or secondary referring hospital. For the 57 unstable patients with known injury time, the median time from injury to presentation at the *CIUSSS de l’Estrie—CHUS* was 115 (52, 198) minutes. Median time to CT scan after arrival at the trauma center was 45 (33, 79) minutes for the 53 unstable patients who had a CT scan within six hours from arrival. Median time to first surgical or angiographic intervention after arrival at the trauma center was 143 (94, 206) minutes among the 24 unstable patients who underwent surgical or endovascular procedures. [Table 6] Emergent surgery or angiography was performed for 12/26 (46%) patients that received early vasopressors and 12/37 (32%) patients that did not receive early vasopressors (p = 0.30). Time between injury and arrival at the *CIUSSS de l’Estrie—CHUS* was longer for patients who transited through referring primary or secondary hospitals compared to patients who were brought directly to the trauma center (243 vs. 61 minutes, p<0.01).

**Table 6:** Care trajectories of hemodynamically unstable patients.

	Overall			Transfer						p-value
				yes			no			
Time (minutes)	n	Median	Q1, Q3	n	Median	Q1, Q3	n	Median	Q1, Q3	
Injury to trauma center	57	115	52, 198	20	243	184, 382	37	61	48, 102	<0.01
Injury to OR* or angiography	24	300	205, 460	9	460	331, 545	14	223	143, 300	<0.01

*OR: operating room

p-values by Wilcoxon Rank-Sum test

### Outcomes

Ten (10/63, 16%) hemodynamically unstable patients and 6/48 (13%) stable patients died during the course of their hospital stay (p = 0.62). Within the hemodynamically unstable group, mortality was significantly higher in patients with concomitant severe TBI (41% vs. 3%, p<0.01). Twenty-seven (27/63, 44%) hemodynamically unstable patients were ultimately discharged home and 14/63 (23%) required transfer to a rehabilitation center. [Table 7]

**Table 7:** Outcomes.

Variable		Hemodynamic Status		p-value	Hemodynamically Unstable Patients		p-value
		Unstable	Stable		Without Severe TBI	Severe TBI	
		n = 62*	n = 48		n = 40	n = 22	
Required surgery or angiography		24	--	--	15	9	0.79
In-Hospital Mortality		10 (16.1%)	6 (12.5%)	0.79	1 (2.5%)	9 (40.9%)	<0.01
Length of ICU Stay (days)		4.0 (2.0, 7.0)	3.0 (2.0, 4.8)	0.02	4.0 (2.3, 5.0)	6.0 (2.0, 12.0)	0.10
Length of Hospital Stay (days)		12.5 (7.0, 27.0)	8.5 (3.3, 14.8)	0.01	12.5 (8.0, 27.8)	15.0 (3.0, 26.8)	0.46
Discharge Disposition	Home	27 (44.6%)	24 (50.0%)	0.57	24 (60.0%)	3 (13.6%)	<0.01
	Rehabilitation	14 (22.6%)	10 (20.8%)	1.00	7 (17.5%)	7 (31.8%)	0.22
	Transfer to another Hospital	8 (12.9%)	4 (8.3%)	0.55	6 (15.0%)	2 (9.1%)	0.70
	Convalescence Home	2 (3.2%)	3 (6.3%)	0.65	1 (2.5%)	1 (4.5%)	1.00
	Long-Term Care	1 (1.6%)	1 (2.1%)	1.00	1 (2.5%)	0 (0.0%)	1.00

*One patient was transferred to another hospital after six hours at the CIUSSS de l'Estrie—CHUS and was excluded from these analyses

Categorical variables are presented as count (%), duration of stay variables as median (Q1, Q3). Proportions are compared using Fisher's exact test, time variables compared using Wilcoxon Rank-Sum test. TBI—traumatic brain injury; severe TBI defined as Glasgow Coma Scale ≤8

## Discussion

The results of this single-center retrospective observational study of early resuscitation practices for trauma are context-specific. The predominant mechanism of injury was blunt trauma and transport delays, due to the geographical spread of the population, were notable. In this cohort of hemodynamically unstable patients, as defined by hypotension or the delivery of resuscitative interventions (i.e. the administration of vasopressors or >2 L of intravenous fluids), patients received significant volumes of intravenous fluids and vasopressors were used in over a third of cases, and more commonly among victims of severe TBI. In contrast, ISS was not associated with vasopressor use.

Our findings highlight that vasopressors may be used more commonly than is recommended in highly cited guidelines. [10] Given that blunt trauma constitutes the predominant mechanism of injury in our population, permissive hypotension could be less beneficial or even cause harm, particularly among victims at high risk for concomitant TBI. [25] The higher incidence of vasopressor use in this study compared to previous reports, ranging between 13 and 26%, [18, 19] may result from our decision to include patients with TBI. We chose to include these patients since the study of hemodynamic resuscitation remains relevant in patients who have both TBI and signs of hemodynamic instability. Moreover, during the early phase of resuscitation (i.e., before secondary and tertiary surveys are completed and imaging becomes available), the importance of cerebral injuries is often unclear since unconsciousness may be explained by a host of other factors ranging from shock to substance abuse. Until the clinical picture becomes clear, patients are likely to be exposed to generic resuscitation protocols. Furthermore, as patients with TBI are both more vulnerable to hypotension and more likely to receive vasopressors, this subgroup is of particular interest for future studies on the role of vasopressors in trauma.

In the province of Quebec, trauma care is overseen by a government body which establishes guidelines for all centers to follow, l’*Institut national d’excellence en santé et en services sociaux* (INESSS). INESSS recommends the use of the PTI rather than other metrics, such as the Shock Index [26] or the Revised Trauma Score [27], in the initial evaluation of trauma patients [28]. Moreover, the early resuscitation of trauma patients in our institution is led by ATLS-trained emergency physicians that act as trauma team leaders in the emergency department. In cases of severe injury, a multidisciplinary trauma team is activated that involves general surgery, anaesthesia and critical care. Resuscitation once a patient leaves the emergency department is usually led either by the anaesthetist in the operating room or by the intensivist in the ICU, with significant input from the surgical team. The extended length of time required for unstable patients in our cohort to reach the operating room sheds light on the challenges of delivering high-quality trauma care in comparably low-volume trauma centers. Trauma systems, through ongoing monitoring of quality and safety metrics, have been shown to improve the delivery of care to trauma populations. [29, 30] Since we did not collect the indication for surgery, it is also possible that some patients were operated for reasons unrelated to hemodynamic instability.

The generalizability of these findings is therefore limited by the single-center design. Notwithstanding, our data are concordant with other reports suggesting that high rates of penetrating trauma are rare outside densely populated urban areas in North America. For example, the prevalence of injuries caused by blunt trauma ranged from 82 to 96% in published reports of trauma cohorts from Germany, [31] Scandinavia, [32] China [33] and across Canada. [34]

Another limitation is that the retrospective design precluded the ascertainment of intentionality. For example, we could only infer the duration of the resuscitation period. When large volumes of blood products were administered, we were unable to determine if the objective was to restore circulating volume or to correct coagulopathies. With vasopressors, we could not determine whether the intention was to correct hypotension or to achieve adequate cerebral perfusion pressures in patients with severe TBI. Lastly, by design, we could not reliably measure associations between resuscitation strategies and clinical outcomes. To avoid indication bias, this can only be measured in a randomized clinical trial.

Strengths of this work include the comprehensive description of resuscitation care delivered to trauma patients in a Canadian regional trauma center, the inclusion of all trauma patients that presented to the institution over the course of one year, and the inclusion of patients who suffered from TBI. Moreover, we defined every variable *a priori* and completed data collection using pre-tested case report forms after validating inter-rater agreement for the main outcome.

In summary, our findings highlight the challenges to delivering high quality trauma care to unstable patients and the importance of trauma care systems. Where patients are at high risk for both hemorrhagic shock and TBI following blunt trauma, measuring the risk-benefit trade-offs of intravenous fluids, vasopressors and permissive hypotension constitutes a priority.

## Supporting information

S1 File Data for analyses. (CSV)
